# Signal-averaged electrocardiography as a noninvasive tool for evaluating the ventricular substrate in patients with nonischemic cardiomyopathy: reassessment of an old tool

**DOI:** 10.3389/fcvm.2024.1306055

**Published:** 2024-04-16

**Authors:** Dinh Son Ngoc Nguyen, Chin-Yu Lin, Fa-Po Chung, Ting-Yung Chang, Li-Wei Lo, Yenn-Jiang Lin, Shih-Lin Chang, Yu-Feng Hu, Ta-Chuan Tuan, Tze-Fan Chao, Jo-Nan Liao, Ling Kuo, Chih-Min Liu, Shin-Huei Liu, Cheng-I Wu, Ming-Jen Kuo, Guan-Yi Li, Yu-Shan Huang, Shang-Ju Wu, Yoon Kee Siow, Jose Antonio L. Bautista, Dat Tran Cao, Shih-Ann Chen

**Affiliations:** ^1^ Division of Cardiology, Department of Medicine, Heart Rhythm Center, Taipei Veterans General Hospital, Taipei City, Taiwan; ^2^ Cardiology Department, University Medical Center, Ho Chi Minh City, Vietnam; ^3^ Institute of Clinical Medicine, National Yang-Ming University, Taipei, Taiwan; ^4^ Department of Cardiology, Taichung Veterans General Hospital, Taichung, Taiwan; ^5^ Department of Cardiology, Serdang Hospital, Selangor, Malaysia; ^6^ Section of Clinical Cardiac Electrophysiology, Heart Institute, St. Luke’s Medical Center – Global City, Taguig City, Philippines; ^7^ Arrhythmia Treatment Department, Cho Ray Hospital, Ho Chi Minh City, Vietnam; ^8^ Department of Medicine, National Chung Hsing University, Taichung, Taiwan

**Keywords:** signal-averaged electrocardiography, nonischemic cardiomyopathy, ventricular arrhythmia, right ventricle, epicardium

## Abstract

**Introduction:**

Signal-averaged electrocardiography (SAECG) provides diagnostic and prognostic information regarding cardiac diseases. However, its value in other nonischemic cardiomyopathies (NICMs) remains unclear. This study aimed to investigate the role of SAECG in patients with NICM.

**Methods and results:**

This retrospective study included consecutive patients with NICM who underwent SAECG, biventricular substrate mapping, and ablation for ventricular arrhythmia (VA). Patients with baseline ventricular conduction disturbances were excluded. Patients who fulfilled at least one SAECG criterion were categorized into Group 1, and the other patients were categorized into Group 2. Baseline and ventricular substrate characteristics were compared between the two groups. The study included 58 patients (39 men, mean age 50.4 ± 15.5 years), with 34 and 24 patients in Groups 1 and 2, respectively. Epicardial mapping was performed in eight (23.5%) and six patients (25.0%) in Groups 1 and 2 (*p* = 0.897), respectively. Patients in Group 1 had a more extensive right ventricular (RV) low-voltage zone (LVZ) and scar area than those in Group 2. Group 1 had a larger epicardial LVZ than Group 2. Epicardial late potentials were more frequent in Group 1 than in Group 2. There were more arrhythmogenic foci within the RV outflow tract in Group 1 than in Group 2. There was no significant difference in long-term VA recurrence.

**Conclusion:**

In our NICM population, a positive SAECG was associated with a larger RV endocardial scar, epicardial scar/late potentials, and a higher incidence of arrhythmogenic foci in the RV outflow tract.

## Introduction

Nonischemic cardiomyopathy (NICM) is a group of diseases affecting the myocardium without significant coronary artery disease. The pathogenesis of NICM can be genetic, inflammatory, toxic, or viral. However, in most cases, its origin is unclear (1). Managing ventricular arrhythmias (VAs) in NICM includes anti-arrhythmic drug therapies, implantable cardioverter–defibrillator implantation, ablation, and surgical intervention to eliminate arrhythmogenic substrates. With the understanding of abnormal ventricular substrates and advancements in three-dimensional systems, radiofrequency catheter ablation (RFCA) has become an optimal strategy for NICM patients with drug-refractory ventricular tachycardias (VTs) (2). Signal-averaged electrocardiography (SAECG), a noninvasive examination to recognize abnormal ventricular conduction, provides essential information for identifying abnormal potentials and assists in the diagnosis of arrhythmogenic right ventricular (RV) cardiomyopathy (ARVC) (3).

Additionally, SAECG, which noninvasively records late potentials of myocardial disease, is useful for risk classification in patients with non-ARVC NICM (4, 5). However, the value of SAECG in predicting the ventricular substrate characteristics in NICM remains unclear. This study aimed to assess the value of SAECG for predicting the locations of arrhythmogenic and diseased substrates in patients with NICM.

## Methods

### Study population

This was a retrospective study of patients who underwent VA ablation at the Heart Rhythm Center, Taipei Veterans General Hospital, between 2012 and 2022. Patients were diagnosed with NICM based on the clinical presentation of heart failure, excluding coronary disease, by echocardiography and angiography (2, 6, 7). Patients diagnosed with NICM and VA who underwent biventricular mapping were enrolled. Patients with baseline ECG showing a bundle branch block pattern or ventricular pacing rhythm were excluded. We also excluded patients diagnosed with ARVC based on medical charts review and Modification of the Task Force Criteria. The indications for catheter ablation included (1) recurrent sustained monomorphic VT refractory to antiarrhythmic drugs and (2) a high burden of ventricular premature complexes (VPCs) and documented nonsustained VT refractory to antiarrhythmic drugs (AADs) in symptomatic individuals. The epicardial approach was considered in selected patients with NICM (2, 8). The endocardial approach was initially done in all the patients. The epicardial mapping was performed if there was one of the following criteria “(1) unmatched endocardial substrate and VT exit, (2) lack of abnormal substrate in the endocardium, (3) failed endocardial ablation, and (4) incomplete VT circuit with endocardial mapping during VT” (9).

This study was approved by the Institutional Review Board of Taipei Veterans General Hospital (approval number 2022-08-018AC).

All patients underwent 12-lead ECG, SAECG, 24-h Holter monitoring, transthoracic echocardiography, coronary artery angiogram, and electrophysiological evaluations. Magnetic resonance imaging (MRI) was performed in patients without any contraindications.

The population was categorized into two groups: Group 1, patients who fulfilled ≥1 SAECG criterion, and Group 2, patients who did not fulfill any SAECG criterion. Baseline patient characteristics, SAECG results before ablation, echocardiography parameters, electrophysiological study, ventricular mapping, and ablation site were assessed.

### SAECG

SAECG is a noninvasive signal processing technique that detects abnormal late potentials in the terminal portion of QRS complexes during sinus rhythm. “The 2010 Revised Task Force Criteria defined positive SAECG as any of the following three criteria: (1) filtered QRS duration: ≥114 ms; (2) terminal QRS duration <40 mV: ≥38 ms; and (3) terminal (last 40 ms) QRS root mean square voltage: ≤20 mV. In our study, all the patients underwent SAECG before undergoing RFCA using a MAC 5500 HD system (GE Healthcare, Freiburg, Germany). The values were recorded when a noise level of 0.3 mV was obtained by averaging 250 beats. The signal-to-noise ratio was 140 dB.” (10).

### Electrophysiology study, substrate mapping, and catheter ablation of ventricular arrhythmias

All patients provided informed consent for the electrophysiological study and ablation. At the beginning of each procedure, all the patients underwent a standardized routine electrophysiological study in a fasting state. All antiarrhythmic drugs, except amiodarone, were discontinued for at least five half-lives before the procedure. 31% of patients used amiodarone before the procedure. Amiodarone was discontinued three days before the procedure in patients without life-threatening ventricular arrhythmias. For patients experiencing frequent episodes of ventricular arrhythmia, amiodarone was continued. If the clinical VA was not spontaneous, rapid ventricular pacing and/or programmed stimulation using three extra stimuli were performed from the RV apex and/or RV outflow tract (RVOT) to induce VA, with and without isoproterenol infusion (1–5 μg/min). The QRS morphologies and cycle lengths of spontaneous and/or induced VA were compared with clinically documented VA (8). All patients underwent three-dimensional electroanatomical mapping of the right and left ventricles (RV and LV) before ablation. An open-irrigated tip ablation catheter was used in all the patients.

Bipolar scar and low-voltage RV and LV areas are areas with peak-to-peak bipolar voltages of <0.5 and <1.5 mV, respectively, and RV and LV unipolar LVZ areas are areas with peak-to-peak unipolar voltages of <5.5 and <8.3 mV, respectively (10, 11). The epicardial scar was considered once bipolar electrogram amplitude less than 0.5 mV, and LVZ as bipolar electrogram amplitude of 0.5–1.0 mV (2). Multielectrode mapping catheters were utilized for mapping in this study such as decapolar, PentaRay (Johnson & Johnson, New Brunswick, NJ), Advisor™ HD Grid Mapping Catheter (Abbott, St. Paul, MN, USA) gride for RV, LV and epicardial mapping before ablation. Both unipolar voltage and bipolar voltage were extracted for analysis. The surface area measurement tool of the three-dimensional system was used to measure the scar regions and LVZ of the chamber. When multiple areas with confluent low voltages were present, we calculated the whole LVZ or scar areas. Each percentage value of LVZ or scar area was calculated by dividing it by the total endocardial or epicardial area. The fill threshold was set to 10 mm for preserved voltages and 5 mm for areas with low-voltage amplitude.

Late potentials (LPs) are the local ventricular potentials occurring after the latter portion of the surface QRS (11).

Arrhythmogenic foci were defined as identified VT isthmus, VA exits, local abnormal ventricular activities, or LPs relevant to the VT/VA (2).

### VT ablation strategy

If VT was stable, activation and/or entrainment mapping was performed to localize the VT isthmus. If the clinical presentation was VPC/nonsustained VT-dominant, VPC triggers were eliminated (2). A substrate-based ablation, which targeted late and fractionated potentials within or around the scar/LVZ, was performed in all patients (2).

Successful ablation was defined as the absence of any spontaneous or inducible VA at the end of the procedure using the same stimulation protocol with or without isoproterenol. Partial success was defined as spontaneous or inducible non-clinical VA after ablation. If clinical VAs still induced at the end of procedure, we considered it as failure ablation (12).

### Follow-up

Patients were followed up for 1, 3, and 6 months within the first year and every 3 months thereafter. Implantable cardioverter-defibrillator (ICD) interrogation and ECG were performed every 3 months. Holter monitoring or event recording was performed at the 3rd, 6th, and 12th months and then at least once yearly after the procedure in patients without an ICD. VT/ventricular fibrillation (VF) recurrence was defined as the recurrence if there was VT/VF on ECG monitoring or ICD recording. VPC recurrence was noted if the VPC burden after ablation was higher than 5000 beats during the 24 h of monitoring or ICD recording.

Patients unable to attend follow-ups at our institution were reviewed at local institutions and underwent telephone consultations for recurrent symptoms and arrhythmia burden.

### Statistical analysis

Continuous variables are expressed as mean ± SD and nominal data as number (*n*) and percentage (%). Baseline characteristics, echocardiographic parameters, and ventricular substrate data of the patients were compared using Student's *t*-test for continuous variables and the *χ*^2^ test for categorical variables, with or without Yates correction or Fisher's exact test. Statistical significance was defined as *p* < 0.05. All the statistical analyses were performed using SPSS Version 27.0 (IBM Corp., Armonk, NY, USA).

## Results

### Baseline characteristics of the participants

The study included 58 patients (39 men, 67.2%) with a mean age of 50.4 ± 15.5 years. The study population was classified into Groups 1 (*n* = 34, 58.6%) and 2 (*n* = 24, 41.4%). Epicardial mapping was achieved in 14 patients (24.1%), including eight patients (23.5%) in Group 1 and six patients (25.0%) in Group 2 (*p* = 0.897). The baseline characteristics of the study participants are presented in Table 1. Patients in Group 1 had a higher prevalence of hypertension (38.2% vs. 4.2%, *p* = 0.003). There were no significant differences between the two groups in other variables such as age, sex, diabetes mellitus, congestive heart failure, ICD implantation, syncope, palpitation, VA characteristics, and LV ejection fraction.

**Table 1:** Baseline characteristics.

Variables	Group 1 *N* = 34	Group 2 *N* = 24	*P* value
Age	53.2 ± 15.7	46.4 ± 14.5	0.100
Male	23 (67.6%)	16 (66.7%)	0.938
Hypertension	13 (38.2%)	1 (4.2%)	0.003
Diabetes mellitus	1 (2.9%)	2 (8.3%)	0.361
CHF	10 (29.4%)	2 (8.3%)	0.051
ICD implantation	7 (20.6%)	1 (4.2%)	0.074
Syncope	11 (32.4%)	7 (29.2%)	0.796
Palpitation	24 (70.6%)	13 (52.4%)	0.200
Shortness of breath	9 (26.5%)	6 (25.0%)	0.900
LVEF (%)	47.4 ± 14.6	52.7 ± 11.6	0.156
LVIDd (mm)	53.0 ± 9.3	50.6 ± 6.0	0.373
LVIDs (mm)	38.2 ± 12.3	34.9 ± 6.8	0.298
LVEDV (ml)	112.6 ± 50.8	87.1 ± 30.3	0.075
LVESV (ml)	61.3 ± 43.8	42.1 ± 23.5	0.111
Sustained VT/VF	21 (61.8%)	9 (37.5%)	0.069

CHF, congestive heart failure; ICD, implantable cardioverter defibrillator; LVEF, left ventricular ejection fraction; RVEF, right ventricular ejection fraction; VT, ventricular tachycardia; VF, ventricular fibrillation; LVIDd, left ventricular end-diastolic diameter; LVIDs, left ventricular end-systolic diameter; LVEDV, left ventricular end-diastolic volume; LVESV, left ventricular end-systolic volume.

### Ventricular substrate and arrhythmogenic potentials

**Figure 1:**
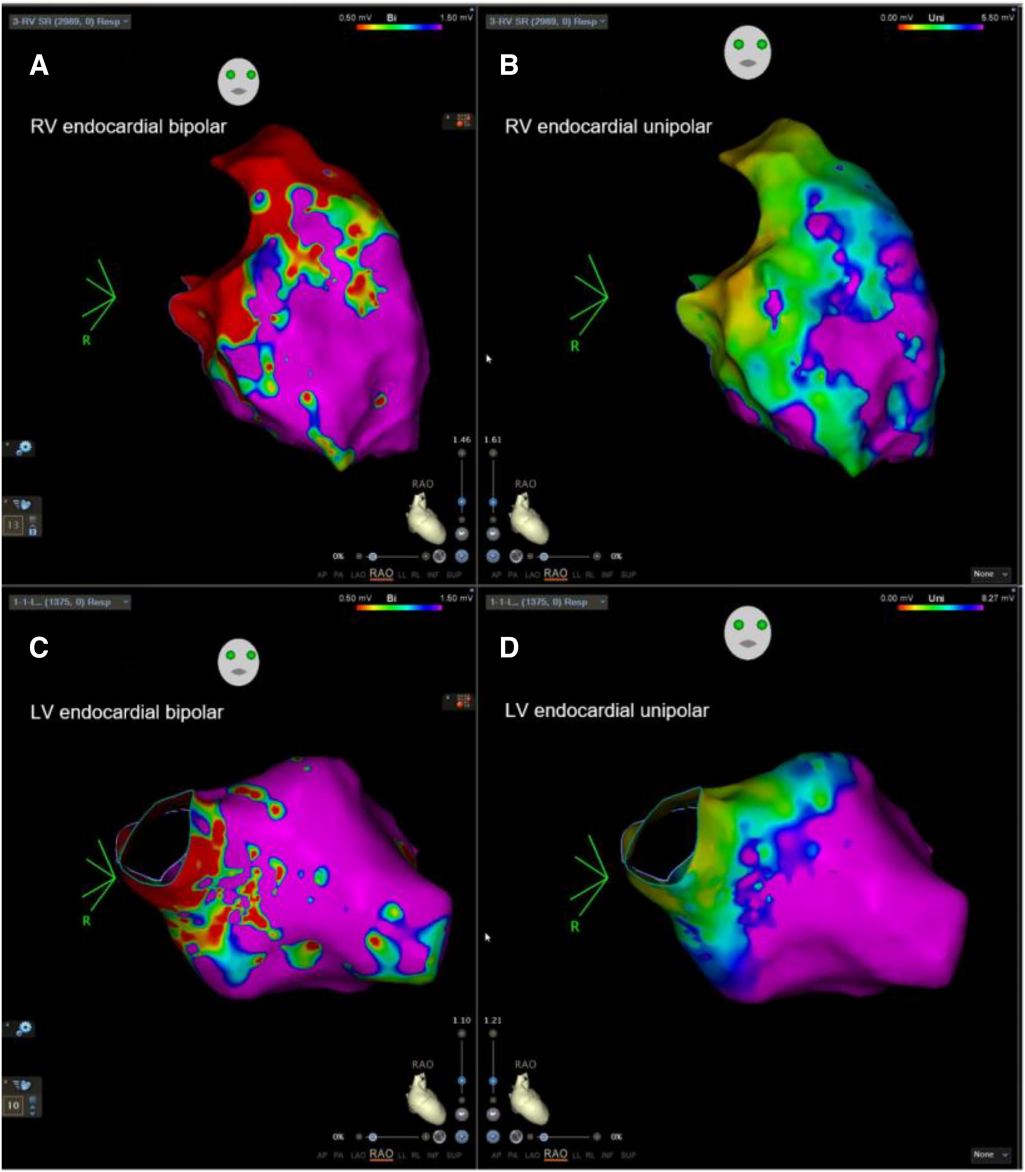
Example of biventricular mapping in a patient with SAECG (+). RV and LV voltage maps of a patient with SAECG (+) showing an LVZ in the RV and LV endocardium. Substrate modification was performed at the RV septum and low septal side of RVOT tract with local abnormal ventricular activity. RV, right ventricle; LV, left ventricle; RVOT, right ventricular outflow tract; LVZ, low-voltage zone; LP, late potentials.

**Figure 2:**
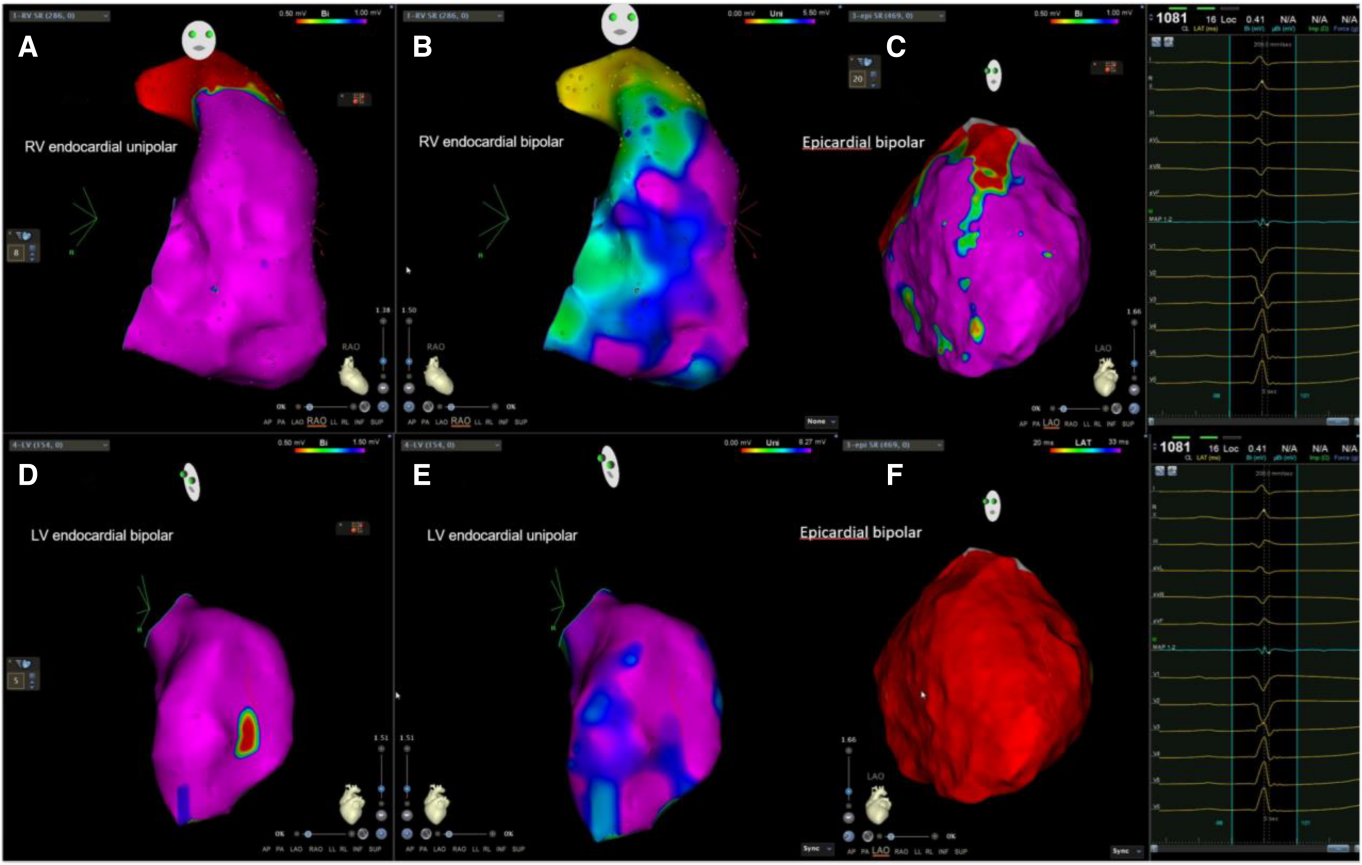
Example of biventricular mapping in a patient with SAECG (−). Voltage map in a patient with SAECG (−); RV, LV, and epicardial substrate mapping showed minimal LVZ and no obvious LPs at the epicardium. Ablation was done at the anterior free wall RVOT and epicardial LV summit. RV, right ventricle; LV, left ventricle; LVZ, low-voltage zone; LPs, late potentials; RVOT, right ventricular outflow tract.

Patients in Group 1 had a significantly longer RV median total activation time (TAT), larger RV unipolar LVZ, and larger RV scar area than those in Group 2 (Table 2, Figures 1,2).

**Table 2:** Endocardial and epicardial ventricular substrate characteristics.

Variables	Group 1 *N* = 34	Group 2 *N* = 24	*p* Value
RV mapping			
RV unipolar median (mV)	6.03 ± 2.10	6.44 ± 2.49	0.497
RV bipolar median (mV)	2.22 ± 1.04	2.79 ± 1.31	0.071
RV TAT (ms)	188.85 ± 70.40	153.96 ± 42.16	0.022
RV area (cm^2^)	222.77 ± 55.51	207.35 ± 53.16	0.293
RV unipolar LVZ area (cm^2^)	51.00 ± 45.33	21.46 ± 31.07	0.008
RV unipolar LVZ percentage (%)	22.63 ± 19.29	10.21 ± 14.42	0.010
RV bipolar LVZ area (cm^2^)	23.46 ± 26.90	12.03 ± 16.54	0.051
RV bipolar LVZ percentage (%)	9.78 ± 9.90	5.92 ± 8.06	0.120
RV bipolar scar (cm^2^)	14.87 ± 20.36	1.73 ± 3.11	0.001
RV bipolar scar percentage (%)	6.01 ± 7.20	0.90 ± 1.59	<0.001
LV mapping			
LV unipolar median (mV)	10.00 ± 4.39	12.37 ± 4.54	0.050
LV bipolar median (mV)	2.47 ± 1.44	3.06 ± 1.08	0.095
LV TAT (mV)	148.38 ± 56.54	147.42 ± 65.97	0.953
LV area (cm^2^)	192.44 ± 63.51	173.28 ± 43.91	0.180
LV unipolar LVZ area (cm^2^)	35.79 ± 45.72	23.96 ± 46.46	0.339
LV unipolar LVZ percentage (%)	19.52 ± 22.57	12.00 ± 22.50	0.216
LV bipolar LVZ area (cm^2^)	18.70 ± 24.80	10.32 ± 20.37	0.179
LV bipolar LVZ percentage (%)	10.00 ± 10.37	5.23 ± 9.03	0.075
LV bipolar scar (cm^2^)	10.09 ± 27.02	1.31 ± 5.36	0.073
LV bipolar scar percentage (%)	4.74 ± 12.861	0.61 ± 2.37	0.076
Epicardial mapping			
Variables	Group 1 *N* = 8	Group 2 *N* = 6	*p* Value
Epi unipolar median (mV)	4.75 ± 1.73	5.45 ± 0.44	0.352
Epi bipolar median (mV)	1.13 ± 0.28	1.92 ± 0.44	0.001
Epi TAT (ms)	249.75 ± 85.41	173.50 ± 42.14	0.069
Epi area (cm^2^)	357.64 ± 175.88	413.75 ± 145.34	0.538
Epi bipolar LVZ area (cm^2^)	117.84 ± 62.68	38.40 ± 25.21	0.009
Epi bipolar LVZ percentage (%)	39.87 ± 25.69	8.98 ± 5.94	0.011
Epi bipolar scar (cm^2^)	14.90 ± 21.61	6.9 ± 13.86	0.445
Epi bipolar scar percentage (%)	2.75 ± 3.92	1.97 ± 3.69	0.712

RV, right ventricular; LV, left ventricular; TAT, total activation time; LVZ, low voltage zone; Epi, epicardial.

**Figure 3:**
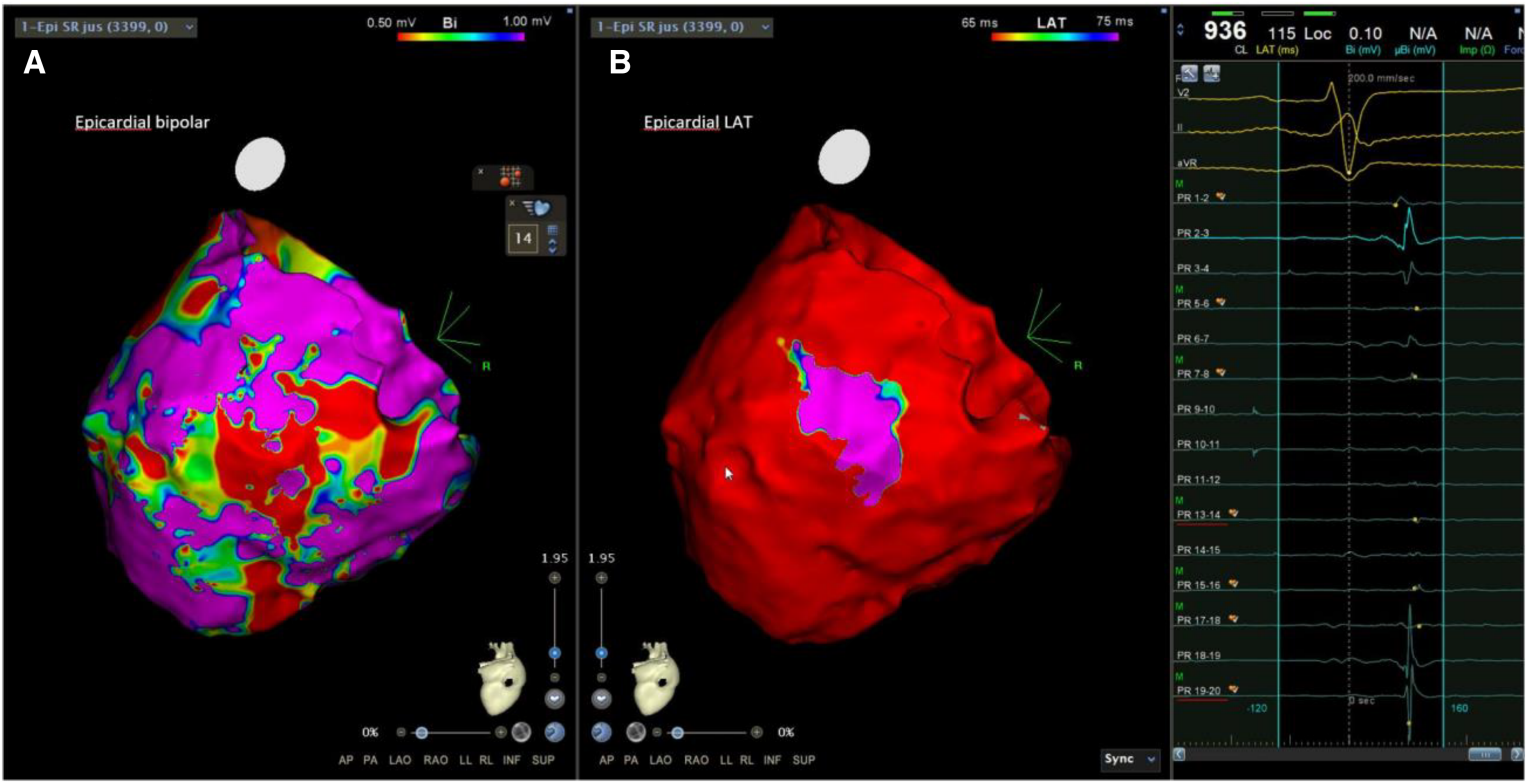
Examples of LPs in patients with SAECG (+). The epicardial substrate map of another SAECG (+) patient showed an LVZ, scar areas, and LPs at the epicardium. LVZ, low-voltage zone; LPs, late potentials.

In the subgroup analysis of 14 patients who underwent epicardial mapping, patients in Group 1 had a lower epicardial bipolar voltage and larger epicardial LVZ than those in Group 2 (Table 2). Group 1 also had a higher incidence of epicardial LPs than Group 2 (50% vs. 0%, *p* = 0.040) (Table 3, Figure 2,3).

**Table 3:** Arrhythmogenic foci with PVC trigger or abnormal electrogram.

Ablation site	Group 1 *N* = 34	Group 2 *N* = 24	*p*
Epicardium ablation	6 (17.6%)	4 (16.7%)	0.922
RV ablation	23 (67.6%)	13 (54.2%)	0.297
LV ablation	28 (82.4%)	19 (79.2%)	0.760
RV and LV ablation	17 (50.0%)	9 (37.5%)	0.346
Multiple endocardial ablation	22 (64.7%)	11 (45.8%)	0.153
Endocardial and epicardial ablation	6 (17.6%)	4 (16.7%)	0.922
RVOT area	17 (50.0%)	4 (16.7%)	0.009
LVOT area	11 (32.4%)	4 (16.7%)	0.179
RV septal area	5 (14.7%)	2 (8.3%)	0.463
RV free wall area	5 (14.7%)	5 (20.8%)	0.543
LV septal area	12 (35.3%)	11 (45.8%)	0.419
LV free wall area	10 (29.4%)	7 (29.2%)	0.984
Crux area	1 (2.9%)	4 (16.7%)	0.067
Endocardial and epicardial late potential			
Variables	Group 1 *N* = 34	Group 2 *N* = 24	*p* Value
Endocardium	10 (29.4%)	7 (29.2%)	0.984
RV endocardial LP	5 (14.7%)	3 (12.5%)	0.810
LV endocardial LP	6 (17.6%)	5 (20.8%)	0.760
Epicardium	Group 1 *N* = 8	Group 2 *N* = 6	
Epicardial LP	4 (50%)	0	0.040

RV, right ventricular; LV, left ventricular; RVOT, right ventricular outflow tract; LVOT, left ventricular outflow tract; LP, late potential; PVC, Premature ventricular complexes.

### Arrhythmogenic area and outcomes of the procedure

The incidence of patients with multiple ablation sites, biventricular ablation, and both endocardial and epicardial ablation did not significantly differ between the two groups (64.7% vs. 45.8%, *p* = 0.153; 50.0% vs. 37.5%, *p* = 0.346; and 17.6% vs. 16.7%, *p* = 0.922, respectively). The incidence of arrhythmogenic areas at the RVOT was significantly higher in Group 1 than in Group 2 (50.0% vs. 16.73%, *p* = 0.009) (Table 3). In addition, the two groups had no significant differences regarding the other ablation sites. After the first procedure, negative inducibility test was achieved in 82.4% of patients in Group 1 and 87.0% in Group 2 (*p* = 0.640). The positive inducibility with non-clinical VAs or ventricular fibrillation occurred in 17.6% of patients in Group 1 and 13.0% in Group 2. 19 patients had late potentials (LPs), and programmed ventricular stimulation (PVS) induced VT/VF in all patients before ablation. The presence of LPs was not significantly associated with the inducibility before and after ablation (Supplementary Table S2).

**Figure 4:**
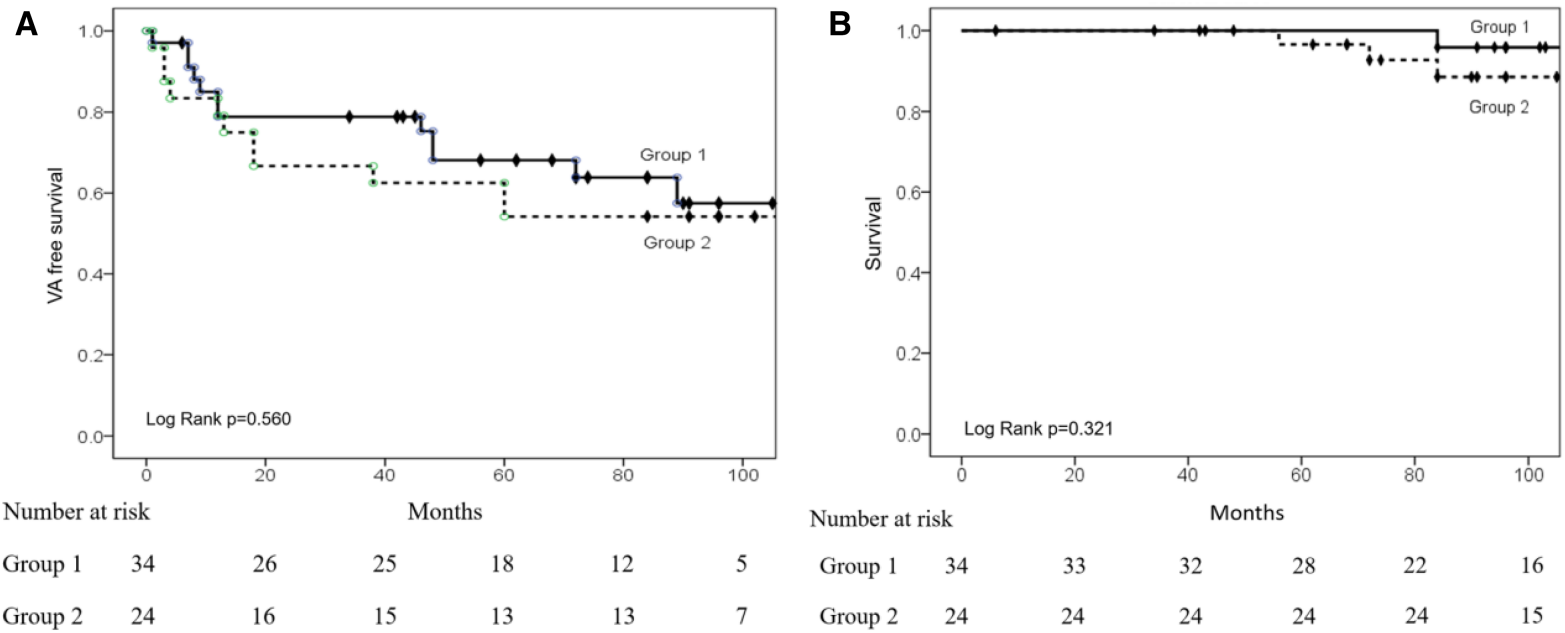
Kaplan–Meier (KM) curves of VA recurrence and survival after ablation. (**A**) The KM curve shows a similar VA recurrence between the two groups. (**B**) KM curve showing similar survival rates between the two groups.

After the procedure, the Kaplan-Meier curve showed no significant difference (log-rank *p* = 0.560) in VA recurrence between the two groups after a mean 61.8 ± 39.7-month follow-up (Figure 4A). There were no significant differences between the two groups regarding VPC/nonsustained VT (29.4% vs. 47.8%, *p* = 0.157) and sustained VT/VF recurrence (8.8% vs. 4.3%, *p* = 0.516).

Four patients died of non-cardiovascular diseases during follow-up. Figure 4B shows the Kaplan-Meier analysis of time to death, with no significant difference between the two groups (log-rank = 0.321) after long-term follow-up.

## Discussion

### Main findings

The present study demonstrated several essential findings in patients with NICM and VT. Patients with a positive SAECG had a longer RV conduction time and more significant areas of RV scarring and epicardial LVZ than those with a negative SAECG. Additionally, the incidence of arrhythmogenic areas in the RVOT was higher in patients with a positive SAECG than in the others.

### Substrate characteristics and SAECG in NICM

SAECG is a non-invasive examination for disclosing the presence of abnormal slow conduction areas in the RV due to ventricular disease (3, 13–15). A positive SAECG was associated with extensive RV and epicardial LVZ in our study. Many studies have confirmed the role of SAECG in assessing ventricular arrhythmogenic potential in ischemic and nonischemic cardiomyopathy. Santangeli et al. found that in ARVC patients, a positive SAECG correlates with LVZ selectively in the RVOT and reflects pathological involvement in the RVOT (13, 16). In the study by Ciconte et al., a positive SAECG was associated with abnormal epicardial electrical activity (17).

The results of our study presented that the correlation between SAECG and the ventricular substrate in patients with NICM, especially the RV scar, epicardial LVZ, and epicardial late potential, was more common in patients with a positive SAECG.

**Figure 5:**
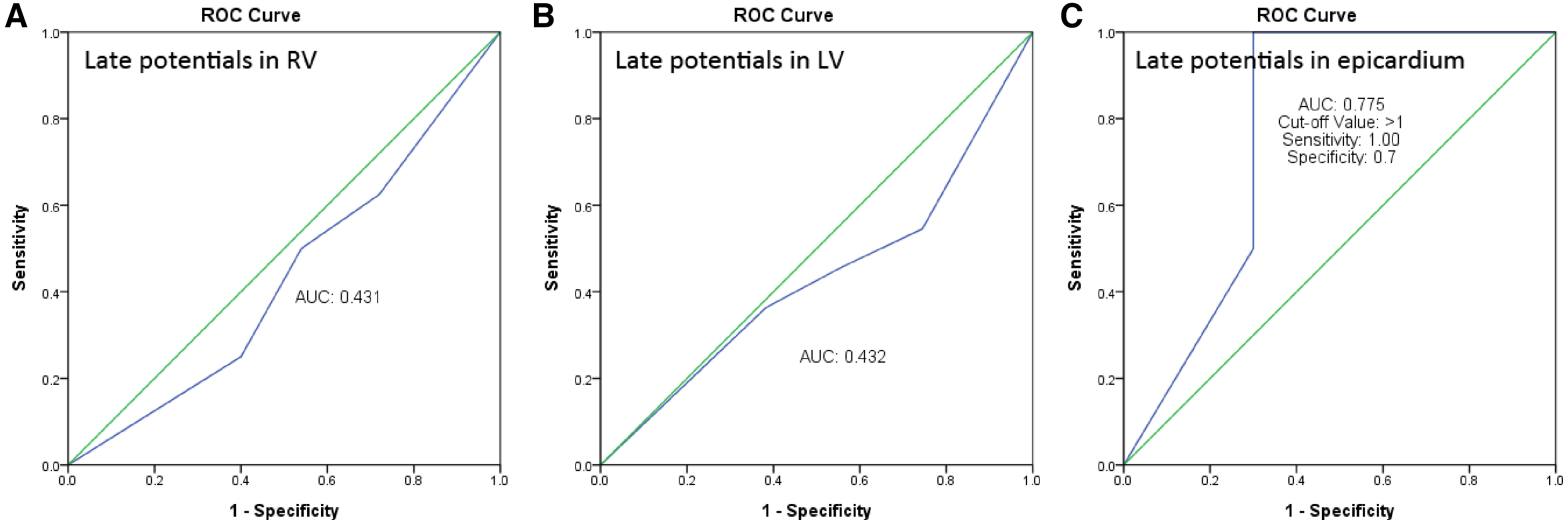
ROC curve analysis for predicting RV, LV and epicardial late potentials.

We performed Receiver Operating Characteristic (ROC) curve analysis, which indicated that the predictive power for endocardial RV late potentials (LP) and left ventricle (LV) LP was limited, with areas under the curve (AUC) of 0.431 and 0.432, respectively. The analysis for predicting epicardial LP demonstrated the optimal cutoff value as greater than 1 positive in the SAECG criteria, exhibiting a sensitivity of 1.0 and specificity of 0.7. Therefore, having more than one positive criterion in the SAECG could effectively predict the presence of LP in the epicardium (Figure 5).

In the present study, the conduction time of RV was more prolonged in Group 1 than in Group 2. This finding might be secondary to the larger RV scar size compared to Group 2 (18). There was no difference between the two groups in any clinical endpoint, such as inducibility or recurrence of arrhythmias post-ablation. The comparable clinical outcomes may be attributed to a similar ablation endpoint in this retrospective single-center study and the relatively small sample size. In this study, conducted at a single center, our goal was to achieve negative inducibility and eliminate all arrhythmogenic substrate in every patient. The similarity in ablation endpoints might account for comparable clinical outcomes despite the differences in right ventricular conduction time.

### Arrhythmogenic substrate and SAECG in NICM

The arrhythmogenic substrates in NICM are primarily located in the basal or perivalvular region of the LV, which is different from ischemic cardiomyopathy with the distribution of abnormal substrates along the territories of the coronary arteries (19).

Previous studies suggest that in patients with NICM, delayed-enhanced imaging and simple ECG analysis allow for predicting anteroseptal or inferolateral scar (20). In the study by Oloriz et al., substantial conduction delay with prolongation of the PR interval, QRS duration or a paced rhythm suggested an anteroseptal scar pattern in NICM. A PR interval of <170 ms and a low voltage, *q* wave, or fragmented QRS in the limb leads is more frequent in patients with inferolateral scar. “A first-line endo-epicardial approach will be more helpful in cases with an inferolateral scar pattern because the arrhythmogenic substrate likely involves the epicardium, and a biventricular endocardial approach should be helpful in cases with an anteroseptal scar, which frequently involves an intramural septal substrate” (20, 21).

There is a difference in the ablation site between patients with NICM and post-myocardial infarction (MI). In patients with post-MI VT, the arrhythmogenic substrate often involves the RV in 11% of patients (22). The underlying VT substrate in patients with NICM can vary, depending on the underlying etiology of NICM.

Conduction disturbances occurred quite common in NICM patients and the presence of the conduction abnormality was associated with an anteroseptal scar pattern in NICM (21). The population of our present study might be different from that of the previous study because of the exclusion of the patients with pacing rhythm or bundle branch block. The difference in population study might explain the arrhythmogenic foci from RVOT incidence were significantly higher in patients with a positive SAECG. In a study conducted by Gatzoulis et al, which showed that “modified late potential criteria such as the presence of two of any of the following three signal averaged parameters: filtered QRS duration > or  =145 ms, low amplitude signal duration > or  =50 ms, root mean square of the last 40 ms of the filtered QRS complex < or  =17.5 microV” is useful for identification of the high risk patients with major degree of conduction defect (23). Modified late potential criteria of SAECG could be use in further studies for accessment ventricular substrates and arrhythmogenic potentials before procedure.

We excluded Arrhythmoginiec Cardiomyophathy patients based on medical charts review and Modification of the Task Force Criteria. Genetic examination was not performed in all patients and this limitation can raise the potential overlap of other cardiomyopathies with Arrhythmogenic Cardiomyopathy.

In our study, the arrhythmogenic foci from RVOT incidence were significantly higher in patients with a positive SAECG and the results of SAECG might not be associated with VA recurrence after ablation in a specific population of NICM. In the ongoing ReCONSIDER study, which introduced a two-step multifactorial approach with noninvasive ECG findings leading to programmed ventricular stimulation (PVS) to detect and protect the truly high-risk population, further study investigating the SAECG in patients with NICM might be warranted for risk stratification (24).

In conclusion, our study provides a noninvasive tool to predict RV endocardial scar or epicardial substrates before mapping and suggests the presence of arrhythmogenic foci at the RVOT area.

## Limitations

First, this was a retrospective study, and cardiac MRI or genetic study were not performed on all patients. It's difficult to analysis the correlation between the presence of late gadolinium enhancement (LGE), LPs and ICD activation. The limitation of genetic evaluation could raise the potential overlap with Arrhythmogenic Cardiomyopathy in our population. Second, this study was limited by its small sample size, which only included few subjects with epicardial mapping. Third, conduction disturbances can result in a positive SAECG. Therefore, the current study enrolled only patients without baseline conduction disturbances. This may limit the patient's population and the results were different from the study conducted by Oloriz et al. The difference in the population study could provide more arrhythmogenic foci in the RVOT. Further studies using the modified SAECG criteria were warranted for our laboratory. Additionally, the present study also enrolled only patients with biventricular mapping, which does not represent the entire NICM population.

## Conclusion

In a selected NICM population, a positive SAECG was frequently associated with RV endocardial scar area, epicardial LVZ area, epicardial LPs, and arrhythmogenic foci at the RVOT. Once patients with NICM have a positive SAECG, the RV approach and epicardial mapping can be considered to eliminate the potential abnormal substrate.

## Data Availability

The original contributions presented in the study are included in the article/**Supplementary Material**, further inquiries can be directed to the corresponding author.
