# Supplementary material for: Signal-averaged electrocardiography as a noninvasive tool for evaluating the ventricular substrate in patients with nonischemic cardiomyopathy: reassessment of an old tool
Source: Front Cardiovasc Med. 2024 Apr 16;11:1306055. doi: 10.3389/fcvm.2024.1306055 (PMC11058987; doi:10.3389/fcvm.2024.1306055)
Supplement: Supplementary file 1 [file Table1.pdf]

Supplementary Table 1: SAECG parameters of population

Variables	Filtered QRS duration (ms)	Terminal QRS duration (ms)	Terminal (last 40 ms) QRS root mean square voltage (ms)
	124.29±26.54	46.07±24.97	25.69±18.71

Supplementary Table 2: inducibility test post-ablation and SAECG results

Variables	SAECG (+)<br>N=34	SAECG (-)<br>N=24	P value
Negative inducibility test	28 (82.4%)	20 (87.0%)	0.898

Abbreviation: SAECG Signal-averaged electrocardiography.

Supplementary Table 3: inducibility test post-ablation and late potentials

Variables	LPs (+)<br>N=19	LPs (-)<br>N=39	P value
Negative inducibility test	18 (94.7%)	31 (79.5%)	0.132

Abbreviation: VT ventricular tachycardia, VF ventricular fibrillation.

Supplementary Table 4. Outcomes of the procedure

Outcomes of procedure		SAECG (+)<br>N=34	SAECG (-)<br>N=24	Total<br>N=58	P
Acute outcome	Success	28 (82.4%)	21 (87.5%)	48 (84.5%)	0.594
	Partial success	6 (17.6%)	3 (12.5%)	9 (15.5%)	
Longterm success		24 (70.6%)	18 (75%)	42 (72.4%)	0.711

Abbreviation: SAECG Signal-averaged electrocardiography.
